# Subtilisin QK-2: secretory expression in *Lactococcus lactis* and surface display onto gram-positive enhancer matrix (GEM) particles

**DOI:** 10.1186/s12934-016-0478-7

**Published:** 2016-05-12

**Authors:** Ruifeng Mao, Kangping Zhou, Zhenwei Han, Yefu Wang

**Affiliations:** State Key Laboratory of Virology, College of Life Sciences, Wuhan University, Wuhan, 430072 People’s Republic of China

**Keywords:** Subtilisin QK-2, Lactic acid bacteria, Secretory expression, Surface display, Gram-positive enhancer matrix, Thrombus

## Abstract

**Background:**

Purified from the supernatant of *Bacillus subtilis* QK02 culture broth, Subtilisin QK-2 is a type of effective thrombolytic reagent that has great exploitable potential. However, the unbearable flavor that occurs with fermentation and the complicated methods that are required to obtain pure products limit the application of this enzyme. Lactic acid bacteria (LAB)-based delivery vehicles are promising as cheap and safe options for medicinal compounds. The secretory expression and surface display using LAB may popularize Subtilisin QK-2 more easily and conveniently with minimal adverse effects.

**Results:**

Subtilisin QK-2 was expressed successfully in two forms using lactic acid bacteria. For the secretory expression in *Lactococcus lactis,* Subtilisin QK-2 was efficiently secreted into the culture using the promoter P_*nisA*_ and signal peptide SP_Usp_. The expression levels were not different in *L. lactis* NZ9000 and NZ3900 without the effect of different selection markers. However, leaky expression was only detected in *L. lactis* NZ3900. The biological activity of this secreted Subtilisin QK-2 was enhanced by modulating the pH of medium to slightly alkaline during induction and by codon optimization of either the entire gene sequence (*qk′*) or only the propeptide gene sequence (*qkpro′*). For surface display onto gram-positive enhancer matrix (GEM) particles, n LysM repeats from the C-terminal region of the major autolysin AcmA of *L. lactis* were fused to either the C-terminus (n = 1, 3, 5) or the N-terminus (n = 1) of the Subtilisin QK-2. These fusion proteins were secreted into the culture medium, and the QK-3LysM was able to bind to the surface of various LAB GEM particles without a loss of fibrinolytic activity. Furthermore, the binding capacity significantly increased with a higher concentration of QK-3LysM. Compared to the free-form Subtilisin QK-2, the QK-3LysM displayed on the surface of GEM particles was more stable in the simulated gastric juice.

**Conclusions:**

Combined with the safety and popularity of LAB, Subtilisin QK-2 may be easily applied worldwide to prevent and control thrombosis diseases.

**Electronic supplementary material:**

The online version of this article (doi:10.1186/s12934-016-0478-7) contains supplementary material, which is available to authorized users.

## Background

Microbial fibrinolytic enzymes have recently attracted greater attention for thrombolytic therapy. As a homolog to Nattokinase (EC 3.4.21.62), Subtilisin QK-2 was purified from the supernatant of *Bacillus subtilis* QK02 culture broth. Mature Subtilisin QK-2 consists of 275 amino acid residues (MW = 27.8 kDa) and shows high efficiency for directly cross-linked fibrin degradation, as determined by SDS-PAGE and the fibrin plate method in vitro [1]. Subtilisin QK-2 inhibits tyrosine nitration induced by nitrite, hydrogen peroxide and hemoglobin in vitro and in vivo and protects human umbilical vein endothelial cells (ECV-304) from damage caused by nitrite and hydrogen peroxide [2]. Additionally, Subtilisin QK-2 significantly inhibits thrombus formation and displays strong thrombolytic activity in mice, and its effect is better than that of the clinically used lumbrokinase [3]. Therefore, Subtilisin QK-2 is a type of effective thrombolytic reagent with great exploitable potential, similar to Nattokinse.

Lactic acid bacteria (LAB), which are widely used in the food industry, are present in the intestines of most animals, including humans. Because many have been granted “generally regarded as safe” (GRAS) status, these bacteria can be used as live vehicles for antigenic, functional proteins or for DNA delivery to the mucosal surface [4]. As the best-studied LAB over the last two decades, *Lactococcus lactis* is an ideal bacteria for the food-grade production of recombinant proteins, and many expression systems have been developed and used in the production of heterologous proteins in *L. lactis* [5]. One of the most powerful expression systems used in LAB is the nisin controlled expression (NICE) system, which is based on the genes involved in the biosynthesis and regulation of the antimicrobial peptide, nisin [4]. Using the NICE system, heterologous proteins can be targeted to a specific cell location, including the cytoplasm, cell surface or extracellular medium. By evaluating the influence of localization on the production yields in *L. lactis,* it was concluded that secretion is preferable to cytoplasmic production [6]. The absence of an outer membrane in LAB makes them particularly attractive for exposing bioactive molecules to the extracellular compartment. Anchoring proteins or peptides onto the surface of LAB is promising in various fields of biotechnological applications [7]. Depending on whether it is genetically modified, surface-engineered LAB can be divided into two types, a genetically modified organism (GMO) and a non-living and non-genetically modified gram-positive bacterial delivery particle, which is also referred to as gram-positive enhancer matrix (GEM). For the GMO type, the expression and display of the passenger protein occurs in the same cell that contains the passenger and anchor fusion gene in the expression plasmid or the genome. By contrast, the expression and display of the fusion protein for the GEM type are separated, such that this type needs two different cells, one cell expresses the fusion protein, and the pretreated LAB cell is combined with the fusion protein to bind the anchor to the surface of the GEM particle using the anchor domain contained in the fusion protein [8]. The expression strain can be yeast or *Escherichia coli* in addition to LAB. The LysM repeat exists in the C-terminal region of the major autolysin AcmA (GenBank: U17696.1) of *L. lactis* and is the anchor domain that is most frequently used to display proteins or peptides on GEM particles [8–12]. Fused with the LysM anchor domain to its C-terminus, α-amylase of *Bacillus licheniformis* is secreted into the culture medium by *L. lactis* NZ9000 and can bind to the surface of trichloroacetic acid (TCA)-pretreated LAB GEM particles without a loss of its functional activity [10]. To promote the application of Subtilisin QK-2, we report the secreted expression of this enzyme in *L. lactis* and surface display onto various LAB GEM particles.

## Results

### Construction of QK-secreting and QK-displaying plasmids

**Fig. 1:**
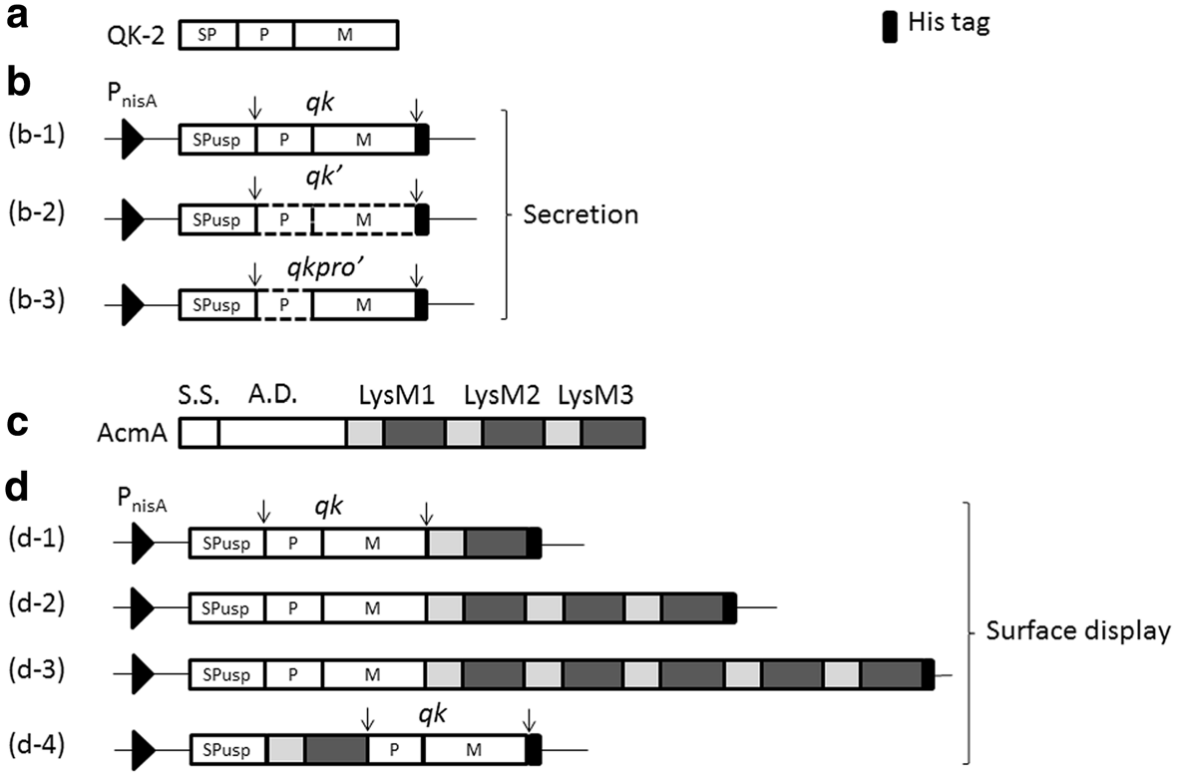
Schematic illustration of proteins or vectors. **a** Schematic illustration of the Subtilisin QK-2 protein. *SP* signal peptide; *P* propeptide; *M* mature peptide. **b** Schematic illustration of secretory expression vectors. *P*_*nisA*_ promoter; *SPusp* secretion signal sequence for *L. lactis*; *qk (b-1)* wild-type gene; *qk′* *(b-2)* the entire gene sequence with codon optimization; *qkpro’* *(b-3)* the only codon-optimized propeptide gene sequence. *(b-1)* contains plasmids pRF02, pRF02H, pRF03 and pRF03H. *(b-2)* indicates plasmid pRF04H. *(b-3)* indicates plasmid pRF05H. **c** Schematic illustration of the AcmA protein. *S.S*. signal sequence; *A.D*. active domain; *LysM* anchoring repeats; **d** schematic illustration of vectors for surface display. *(d-1)*, *(d-2)*, *(d-3)* and *(d-4)* indicate plasmids pRF06H, pRF07H, pRF08H and pRF09H, respectively. pRF02 and pRF02H were selected by the complementation of the *lacF* gene and others were selected by the chloramphenicol resistant gene. *H* his-tag

All plasmids constructed in this study are shown in Fig. 1. All plasmids contain the Usp45 signal peptide sequence (*sp*_Usp_), which enables protein secretion, and is driven by the inducible promoter P_*nisA*_. For secretion of the free Subtilisin QK-2, the wild *qk*, the codon-optimized *qk′* and the only optimized propeptide sequence *qkpro′* (a gene sequence alignment diagram can be found in Additional file 1: Figure S1), were included as shown in Fig. 1b. The LysM repeats from the *acmA* of *L. lactis* MG1363 were fused with the *qk* gene to display this serine protease onto the surface of LAB GEM particles. As shown in Fig. 1d, the nLysM repeats were fused either to the C-terminus (n = 1, 3, 5) or the N-terminus (n = 1) of Subtilisin QK-2.

### Secretory expression of free Subtilisin QK-2

**Fig. 2:**
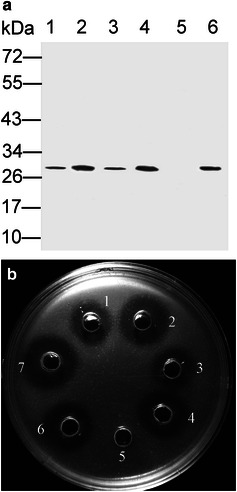
Western blotting analysis (**a**) and fibrinolytic activity analysis (**b**) of Subtilisin QK-2 expressed in *L. lactis*. *1* five times concentrated supernatant of noninduced *L. lactis* NZ3900 (pRF02H); *2* supernatant of induced *L. lactis* NZ3900 (pRF02H); *3* five times concentrated supernatant of noninduced *L. lactis* NZ3900 (pRF03H); *4* supernatant of induced *L. lactis* NZ3900 (pRF03H); *5* five times concentrated supernatant of noninduced *L. lactis* NZ9000 (pRF03H); *6* supernatant of induced *L. lactis* NZ9000 (pRF03H); *7* supernatant culture of the *Bacillus subtilis* QK02. The protein amounts analyzed in western blot were three times that of the fibrinolytic activity analysis

**Fig. 3:**
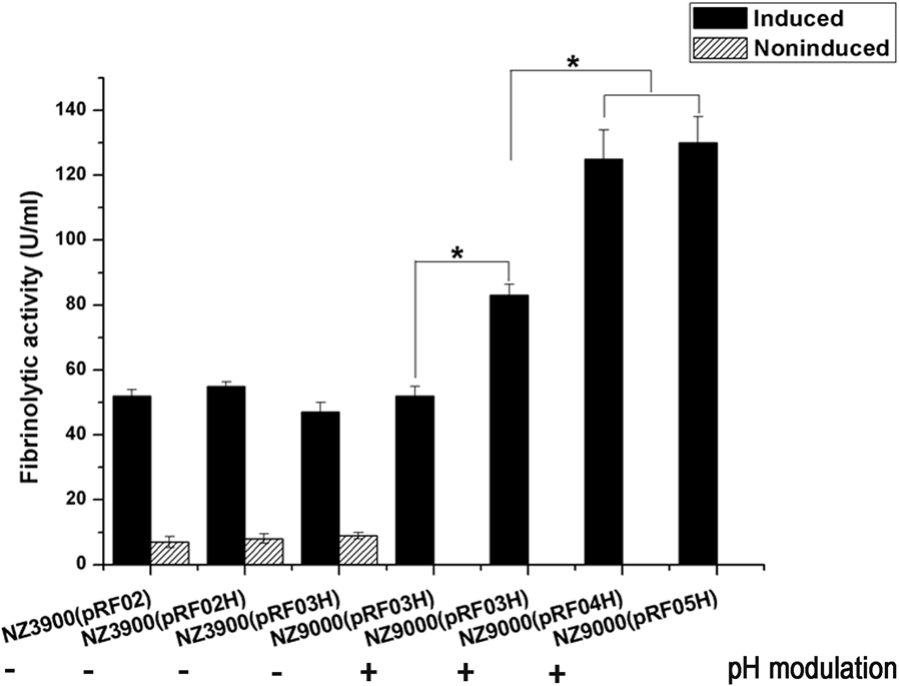
Fibrinolytic activity analysis of different recombinant *L. lactis* strains under the indicated induction conditions. pH modulation indicates that addition of NaOH into the medium at 3 h after induction; (−) meaning without pH modulation, (+) meaning with pH modulation. All data presented were mean ± SD of three replicate experiments (*p < 0.05)

Under the optimized induction conditions (Additional file 1: Figure S2), nisin (70 ng/ml) was added for induction when the OD_600_ of the culture reached approximately 0.5. Then, the culture was incubated at 30 °C for 8 h. A specific band of 28 kDa (identical to the molecular mass of mature Subtilisin QK-2) was detected by western blotting analysis of the supernatant of the induced culture (Fig. 2a) but not in the intracellular lysate (data not shown). The resulting fibrinolytic activity reached approximately 52.3 urokinase units per milliliter culture (Figs. 2b, 3). By testing the fibrinolytic activity of the supernatant culture and the intracellular lysate, secretion efficiency was evaluated, and approximately 96 % of the Subtilisin QK-2 was secreted into the medium. Interestingly, the specific protein bands in the western blot and clearing zones in the fibrinolytic activity analysis were detected not only in the supernatant of the induced culture but also in the non-induced culture of *L. lactis* NZ3900 (pRF02/pRF02H/pRF03H) (Figs. 2, 3). However, Subtilisin QK-2 was detected only in the induced *L. lactis* NZ9000. Under the optimized induction conditions, the expression levels in NZ9000 and NZ3900 were approximately the same, regardless of the selection method used (Fig. 3). By comparing the fibrinolytic activity of the protein samples with the His-tag or untagged proteins (Fig. 3), it was concluded that the His-tag did not interfere with the production or activity of this expressed protein.

### Effect of pH modulation on Subtilisin QK-2 expression

Subtilisin QK-2 is an alkaline protease, and the pH of the medium may play an important role in the expression of this protease. To regulate the pH of the medium, NaOH was added once at 1, 2, or 3 h after induction. The early addition of NaOH (at 1 or 2 h) has an inhibitory effect on growth, as determined by the OD_600_ readings (data not shown). By adding NaOH at 3 h after induction, the ending pH of the medium was 7.3–7.9, and a higher fibrinolytic activity (*p* < 0.05) was detected as shown in Fig. 3.

### Codon usage analysis and effect of codon optimization

The results of the codon usage analysis showed that there are only 23 “low” (10–20 %) usage frequency codons and no “very low” (<10 %) usage frequency codons in the codons of the *qk* gene, as defined in Methods; and that the percentage of “unfavorable” codons is only approximately 6.5 % (23 in 353). Of these 23 rare codons, 11 codons appear in the first 106 codons, which encode the propeptide sequence. Both the wild-type Subtilisin QK-2 gene (*qk*) and the codon-optimized gene (*qk′* and *qkpro′*) were expressed in the same way, and the results were used to study the effect of codon optimization on heterologous expression in *L. lactis*. Compared to the wild-type Subtilisin QK-2 expressed in *L. lactis* NZ9000 (pRF03H), stronger fibrinolytic activity (*p* < 0.05) was detected in the induced culture of *L. lactis* NZ9000 (pRF04H) and NZ9000 (pRF05H) (Fig. 3). The fibrinolytic activity detected in *L. lactis* NZ9000 (pRF04H) and NZ9000 (pRF05H) was similar (Fig. 3). Although the bioactivity of the expressed Subtilisin QK-2 was improved by pH modulation or codon optimization, no visible specific protein band was observed by SDS-PAGE analysis, and the protein bands detected in the western blotting analysis, even for the 50-fold concentrated samples, were comparable to these with no pH modulation or codon optimization (data not shown).

### Expression and characterization of fusion proteins

**Fig. 4:**
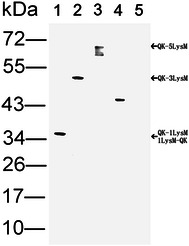
Western blotting analysis of the fusion proteins from the induced supernatant. *1* cells harboring pRF06H expressing QK-1LysM; *2* cells harboring pRF07H expressing QK-3LysM; *3* cells harboring pRF08H expressing QK-5LysM; *4* cells harboring pRF09H expressing 1LysM-QK; *5* cells harboring pNZ8048, negative control. The *right arrows* indicated the expected size, respectively

To use the anchor ability of the LysM repeat, four different plasmids were constructed and transformed into *L. lactis* NZ9000. After induction with no pH modulation, the supernatant was used for western blotting analysis and the results are shown in Fig. 4. All C-terminus fusion samples (QK-1LysM, QK-3LysM, QK-5LysM) showed immunoreactive protein bands of 35, 51 and 66 kDa, respectively, which were the expected size of the recombinant proteins. However, degradation was observed in the protein samples from *L. lactis* NZ9000 (pRF08H), which contained five LysM repeats (QK-5LysM). For the N-terminus fusion protein sample (1LysM-QK), a 44 kDa protein band was detected, which was larger than the expected size of 35 kDa. Analysis of the fibrinolytic activity of these fusion proteins showed that only the C-terminus fusion samples maintained their enzymatic activity; of these three fusion proteins, the enzymatic activity of QK-1LysM and QK-3LysM was approximately 85.7 and 82.4 %, respectively of the free form Subtilisin QK-2 expressed in *L. lactis* NZ9000 (pRF03H), and the activity of QK-5LysM was significantly decreased (Table 1). Therefore, the binding assay was performed with the QK-1LysM and QK-3LysM fusion proteins.

**Table 1:** Fibrinolytic activity of different form QK

Protein	Activity (U/ml)	Percentage (%)
Subtilisin QK-2	53.1	100
QK-1LysM	45.5	85.7
QK-3LysM	43.8	82.4
QK-5LysM	8.3	15.6
1LysM-QK	0	–

### Binding of QK-1LysM and QK-3LysM to LAB GEM particles

**Fig. 5:**
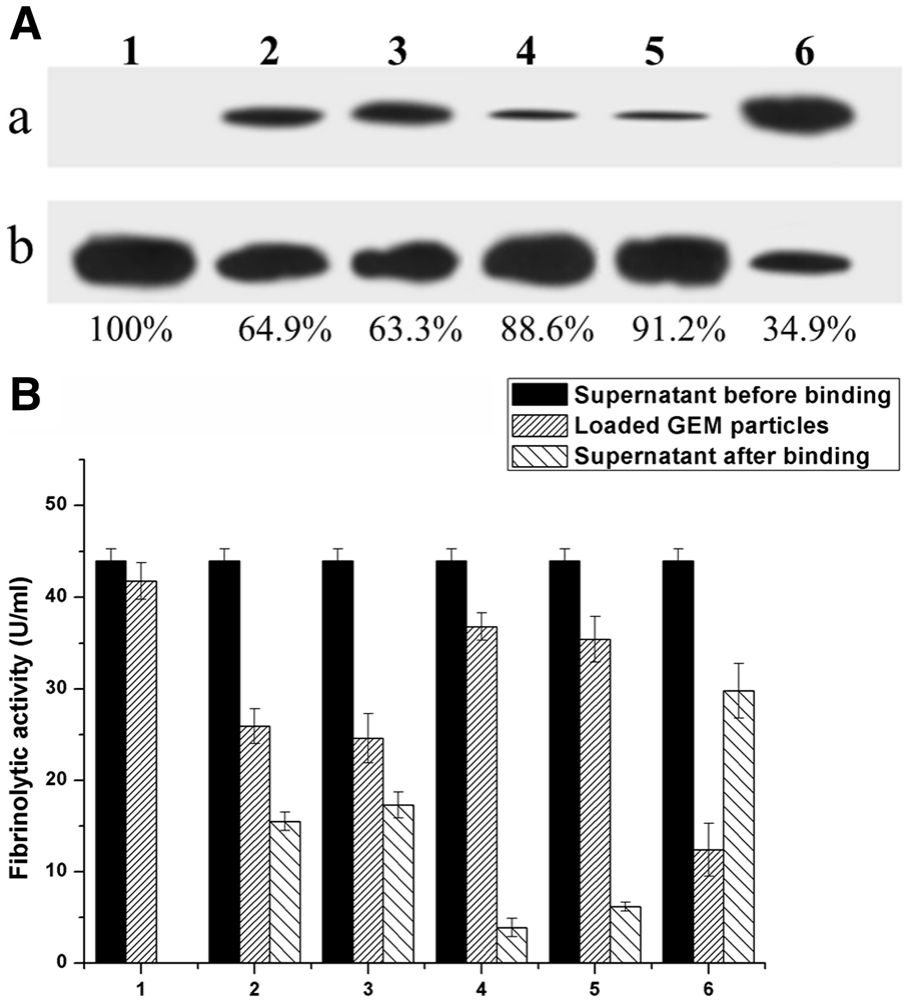
Analysis of the binding efficiency of QK-3LysM. **A** Western blot analysis of the culture supernatant after binding assay (*a*) and the QK-3LysM loaded LAB GEM particles (*b*); the relative binding rate is shown for each LAB GEM particle. **B** Fibrinolytic activity analysis of QK-3LysM before and after binding assay; all data presented were mean ± SD of three replicate experiments. *1* *L. lactis* MG1363; *2* *L. casei*; *3* *L. paracasei*; *4* *S. thermophilus*; *5* *L. bulgaricus* ATCC 11842; *6* *L. plantarum* ST-III

After incubation with 5 ml cell-free culture medium containing QK-1LysM or QK-3LysM, loaded GEM particles were washed and used for western blotting analysis and measure of fibrinolytic activity, as described in the “Methods” section. The GEM samples that were not incubated with the fusion protein or for those incubated with the free Subtilisin QK-2 exhibited no protein bands (data not shown). Both fusion proteins bound to the GEM particles; however, the binding of QK-1LysM was poor because a considerable amount of this fusion protein remained in the culture medium after binding, as shown in Additional file1: Figure S3. By contrast, the protein amounts of the QK-3LysM bound to various LAB GEM particles were higher as determined by western blot and analysis of the fibrinolytic activity (Fig. 5). The binding efficiency calculated by western blot and analysis of the fibrinolytic activity was similar for the corresponding LAB GEM particles. The results show that the binding capacity of the *L. lactis* MG1363 particles is about 100 %, and the relative percentages for the other LAB GEM particles are also shown. Approximately >90 % of the activity was retained after this fusion protein bound to LAB GEM particles (Fig. 5B). Successful binding was also confirmed by immunofluorescence microscopy, as shown in Additional file 1: Figure S4. When the LAB particles were incubated with a high concentration of QK-3LysM, the binding capacity was significantly increased (data not shown).

### Stability in simulated gastric juice

**Fig. 6:**
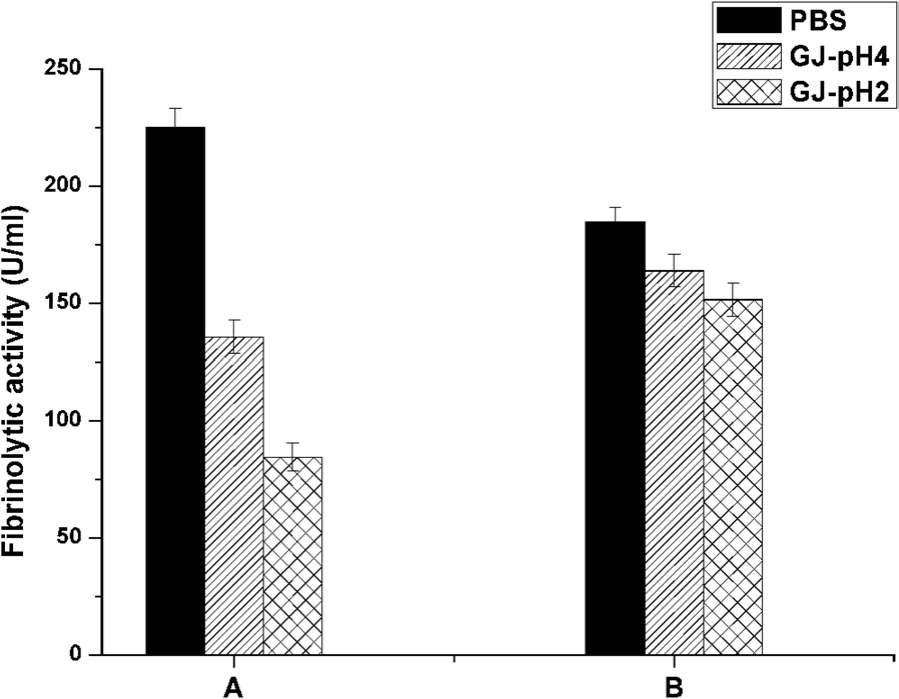
Stability analysis of different form of Subtilisin QK-2 in simulated gastric juice. The free form Subtilisin QK-2 (*A*) and QK-3LysM binding to the surface of *L. lactis* MG1363 GEM particles (*B*) were incubated with simulated gastric juice of pH 4.0 (GJ-pH 4) or pH 2.0 (GJ-pH 2). The samples incubated with PBS (pH 7.0) were used as positive control. All data presented were mean ± SD of three replicate experiments

Simulated gastric juice (GJ) was used to test the stability of the free form Subtilisin QK-2 and the QK-3LysM displayed onto the surface of *L. lactis* MG1363 GEM particles. As shown in Fig. 6, the reduced activity of the loaded MG1363 GEM particles was less than 18 %, even after 30 min at pH 2.0, and the reduction was greater for the free form Subtilisin QK-2, which lost 63 % fibrinolytic activity compared to the positive control (Fig. 6).

## Discussion

Fibrinolytic enzymes derived from microorganisms have attracted more interest in thrombolytic therapy than typical thrombolytic agents, which have drawbacks, including the undesirable side effects, low specificity toward fibrin, and high cost [13]. Subtilisin QK-2 is a subtilisin-family serine protease, and previous studies have revealed that it is a type of effective thrombolytic reagent with highly exploitable potential [1–3]. In this study, Subtilisin QK-2 was first successfully expressed in two forms, including secretory expression and LAB GEM particles-surface display, using the NICE system in *L. lactis*.

Nattokinase is expressed in *B. subtilis* [14] and *E. coli* [15, 16]; however, complicated purification processes must be performed before the expressed enzyme can be applied for clinical use. Although the intracellular [17] and intercellular [18] expression of Nattokinase in *L. lactis* was studied, the recombinant strains contained antibiotic (chloramphenicol and erythromycin, respectively) resistance genes and thus, do not adhere to the real “food-grade” definition [5]. Without using antibiotics, Subtilisin QK-2 was expressed in *L. lactis* NZ3900 (pRF02), which was selected by the complementation of the *lacF* gene in this study, which allowed it to meet the food-grade requirements.

Leaky expression of the *qk* gene from the *nisA* promoter was observed in the recombinant strain *L. lactis* NZ3900. It has been proposed that this leaky expression is a property of the NICE system itself [19]. However, the reason for this phenomenon is unknown and requires further study.

Under the optimized induction conditions, the expression levels in *L. lactis* NZ3900 (pRF02H or pRF03H) and NZ9000 (pRF03H) were similar, regardless of the selection marker used. After 6 and 8 h of induction, the cell growth and production of Subtilisin QK-2 began to decrease, respectively (Additional file 1: Figure S2). However, it is not appropriate to compare the production levels after a long induction time in *L. lactis* because the plasmid stability may not be a key factor [20] that interferes with the production of Subtilisin QK-2.

As an alkaline protease, the optimal pH of Subtilisin QK-2 is approximately 8.5, and it is unstable in acidic environment. Because of the production of lactic acid during fermentation, the pH of the *L. lactis* culture decreases. During the fermentation of the recombinant *L. lactis* NZ9000 (pRF03H) strain, the pH decreased to 5.4, which may affect the expression of Subtilisin QK-2. This is confirmed by regulating the pH of the medium with NaOH. With a slightly alkaline (pH 7.3–7.9) medium, Subtilisin QK-2 had a stronger bioactivity (Fig. 3), which may be attributed to better folding as a result of the addition of NaOH. The same effect of the pH of the medium on the bioactivity of SCI-57 was reported previously [21].

A previous report [22] suggested that codon optimization strategies could be applied to expression systems with LAB as producer strains. In this study, a stronger fibrinolytic activity was found using codon optimization. Furthermore, codon optimization of both the entire sequence and the propeptide sequence only has the same effect (Fig. 3). However, no positive effect of the codon optimization on the yield of this enzyme was detected; and this may be attributed to the low expression level of QK or the low percentage of rare codons (approximately 6.5 %). Optimization of these 11 rare codons existed in the propeptide sequence may improve the activity of the propeptide and then promote the mature polypeptide to fold into the proper conformation, which may be more active or stable, ultimately leading to the same effect as codon optimization of the entire sequence. This also confirms the important role of the propeptide played in the folding process of the mature peptide [23]. Moreover, this finding may offer a new and less expensive method for codon optimization with a target protein similar to Subtilisin QK-2.

The display level of traditional surface display systems, which are based on the expression of proteins on the producer cell surface, is largely dependent on the efficiency of expression and translocation, and the host strain is a genetically modified organism [24]. However, the use of GMOs in applications that involve uncontrolled release into the environment, such as foods and vaccines, is less desirable or at least under debate [10]. To circumvent these problems, gram-positive enhancer matrix (GEM) particles can be used as substrates to bind externally added heterologous proteins by using a high-affinity binding domain. As the most often used binding domain, the LysM repeat exists in the C-terminal region of the major autolysin AcmA of *L. lactis* and was used in this study to display Subtilisin QK-2 onto various LAB GEM particles. For the N-terminus fusion (1LysM-QK), the detected protein band was larger than expected (Fig. 4), and the excess size was equal to the propeptide which should be removed during the translocation process of mature Subtilisin QK-2. Therefore, the propeptide was not cleaved because of the LysM repeat located between the signal peptide and the propeptide. This non-cleaved propeptide resulted in the loss of fibrinolytic activity for this fusion protein. By contrast, the LysM repeats fused to the C-terminus of Subtilisin QK-2 had no influence on the cleavage of the propeptide, and the fusion proteins were expressed at the expected size (Fig. 4) and retained their fibrinolytic activity (Table 1). The fibrinolytic activity of QK-1LysM and QK-3LysM was comparable (85.7 and 82.4 %, respectively) to the free Subtilisin QK-2 contained in the induced culture medium, which indicated that the added anchor domain (1 or 3 LysM repeats) had little impact on the conformation of QK. However, the production of the QK-5LysM fusion protein was lower and highly degraded (Fig. 4). The degradation may be related to the activity of endogenous surface housekeeping proteases, such as HtrA [25], and a greater number of LysM repeats could be more easily targeted compared to one or three repeats. In addition to degradation, the loss of fibrinolytic activity for the QK-5LysM may be attributed to the stereo-hindrance effect of a larger anchor domain. To protect the fusion proteins from degradation, *htrA*-deficient strains therefore represent possible solutions.

After incubation, QK-1LysM was able to bind to GEM particles; however, the binding was poor. With an increase in the number of LysM repeats, a clear increase in the binding affinity was observed in the binding assay of the QK-3LysM fusion protein. This finding is consistent with a previous report [10], in which potent binding to lactococcal GEM particles was obtained using two identical AcmA LysM domains. The binding capabilities of all LAB GEM particles were comparable (Fig. 5) and were higher than those of the natural LAB cells without chemical treatment (data not shown). This is because the binding of LysM-type repeats to peptidoglycans is hindered by other cell wall constituents that exist in the natural cell surface [12]. Most importantly, the binding capacity increased with a higher concentration of QK-3LysM fusion proteins, which improved the fibrinolytic activity of the same volume loaded onto LAB GEM particles. Compared to the free Subtilisin QK-2, the QK-3LysM displayed onto the surface of GEM particles was more stable in simulated gastric juice (Fig. 6). This indicated that the loaded GEM particles can protect Subtilisin QK-2 from degradation and retain considerable functionality after oral administration. Bound to LAB GEM particles, QK-3LysM does not need complex purification process and can be applied to oral administration without any additional adjuvant. Therefore, the lower cost of producing subtilisin loaded LAB GEM particles would contribute to their widespread application. The current administration method of other thrombolytic agents (such as Urokinase and Heparin) is injection and this route is prone to a number of problems and causes huge physical and psychological pressure for patients [26], compared to the gastric delivery of the subtilisin bound to LAB GEM particles.

The fibrinolytic enzyme Subtilisin QK-2 was successfully expressed in the potential probiotic, *L. lactis*. For the secretory expression of Subtilisin QK-2, to avoid potential complications with induction in vivo, it may be possible to preinduce *L. lactis* NZ3900 (pRF02) with nisin prior to oral administration. Treating *L. lactis* with a 1-h pulse of nisin can induce protein secretion for 10 h [27]. Then the recombinant strains can secrete this enzyme in the intestinal tract. Furthermore, *L. lactis* NZ3900 (pRF02) can secrete Subtilisin QK-2 without nisin induction, and this leaky expression may be considered a type of constitutive expression. To circumvent the use of a genetically modified organism and the degradation of free Subtilisin QK-2, the QK-LysM fusion protein (without His-tag) loaded LAB GEM particles can be used for oral administration without additional adjuvants. The highest binding capacity for lactococcal GEM particles is approximately 10^6^ molecules per cell [10]; therefore, a much higher surface density can be obtained by incubation with the culture medium containing a higher concentration of fusion proteins. Additionally other functional proteins can be displayed simultaneously onto the same GEM particles by incubation with culture medium containing different LysM fusion proteins.

## Conclusions

Subtilisin QK-2 was successfully expressed using lactic acid bacteria in two forms, secretory expression and surface display. For secretory expression, both pH modulation and codon optimization improved the bioactivity of this enzyme. In particular, no antibiotic was used in the recombinant strain *L. lactis* NZ3900 (pRF02), which attained the “food-grade” standard. For surface display, the fusion proteins retain fibrinolytic activity with the anchor domain LysM fused to the C-terminus, but not the N-terminus, of Subtilisin QK-2, and the QK-3LysM fusion protein performs best in binding assay. Compared to the free form, Subtilisin QK-2 loaded onto LAB GEM particles is more stable in simulated gastric juice. Using LAB, Subtilisin QK-2 may become popularized more easily and conveniently with minimal adverse effects, and it is a promising strategy for the prevention and control of thrombosis with the potential for widespread application.

## Methods

### Bacterial strains, plasmids, and culture conditions

The bacterial strains and plasmids used in this study are given in Table 2. Strains *L. plantarum* ST-III [28], *L. lactis* NZ9000 [20], and NZ3900 [20] and plasmids pNZ8048 [29] and pNZ8149 [30] were kind gifts from Prof. Shanjing Yao, College of Materials Science and Chemical Engineering, Zhejiang University. *L. delbrueckii* subsp*. Bulgaricus* ATCC 11842 [31] was a kind gift from Xiaoyan Liu, School of Life Science, Huaiyin Normal University. *E. coli* TOP10 (Invitrogen; Carlsbad, CA, USA) and *B. subtilis* QK02 (CCTCC, M203078) strains were grown in Luria–Bertani medium with shaking at 37 °C. *L. lactis* strains were grown statically in M17 medium (Merck) at 30 °C in tightly capped flasks supplemented with 0.5 % glucose (GM17) or 0.5 % lactose (LM17). *Lactobacillus* strains and *S. thermophilus* were grown in MRS broth (Merck) at 37 °C without aeration. Elliker broth [32] was used for the selection of lac^+^*L. lactis* NZ3900 colonies. For plasmid selection in *E. coli* and *L. lactis*, chloramphenicol (Sigma) was added at 5 μg/ml.

**Table 2:** Bacterial strains and plasmids

Strain or plasmid	Relevant properties	Reference or source
Strains		
*E. coli* TOP10	Cloning host	Invitrogen
*B. subtilis* QK02	QK producing bacterium, wild strain	CCTCC
*L. lactis* MG1363	Subsp. *cremoris*, plasmid-free	Our lab
*L. lactis* NZ9000	Derivative of MG1363 with integration *nisRK* into chromosome	[19]
*L. lactis* NZ3900	Derivative of NZ3000 with integration *nisRK* into chromosome; ∆*lacF*	[19]
*L. bulgaricus* ATCC 11842	Type strain	[37]
*L. plantarum* ST-III	Wild strain	[34]
*L. casei*	Wild strain	Our lab
*L. paracasei*	Wild strain	Our lab
*S. thermophilus*	Wild strain	Our lab
Plasmids		
pUC19-qk′	Ap^R^, pUC19 containing the optimized gene *qk*′	TsingKe
pNZ8048	Cm^R^, P_*nisA*_, *L. lactis* expression vector	[35]
pNZ8149	lacF, P_*nisA*_, *L. lactis* food grade expression vector	[36]
pRF01	lacF, pNZ8149 derivate containing *sp*Usp downstream of P_*nisA*_	This study
pRF02	lacF, pRF01 with *qk* downstream of *sp*Usp	This study
pRF02H	lacF, pRF02 containing *qk* with a His-tag	This study
pRF03	Cm^R^, pNZ8048 derivate carrying *sp*Usp-*qk* downstream of P_*nisA*_	This study
pRF03H	Cm^R^, pRF03 containing *qk* with a His-tag	This study
pRF04H	Cm^R^, pRF03 containing *qk’* with a His-tag	This study
pRF05H	Cm^R^, pRF03 containing *qkpro’* with a His-tag	This study
pRF06H	Cm^R^, pRF03 derivate containing one LysM with a His-tag from *acmA* fused to the 3′ end of *qk*	This study
pRF07H	Cm^R^, pRF06H derivate containing three LysM	This study
pRF08H	Cm^R^, pRF06H derivate containing five LysM	This study
pRF09H	Cm^R^, pRF03H containing one LysM from *acmA* between *sp*Usp and *qk*	This study

### DNA manipulation and transformation

Combined with lysozyme, genomic DNA and/or plasmids were extracted from *B. subtilis* and *L. lactis* strains using a rapid bacterial genomic DNA isolation kit (Sangon Biotech, Shanghai, China) and a rapid mini plasmid kit (Tiangen Biotech, Beijing, China), respectively. To study the effect of codon bias on the expression of Subtilisin QK-2 in *L. lactis*, codon optimization was performed using the JCat program [33] according to the protein sequence of Subtilisin QK-2 (GenBank: AJ579472.2). The codon-optimized gene of Subtilisin QK-2 (*qk′*, GenBank accession number KT725198) was synthesized by TsingKe Biological Technology (Wuhan, China) and was ligated into the pUC19 plasmid, resulting in pUC19-qk′. On the basis of the *qk*′, *qkpro*′ (GenBank accession number KT991841) containing the codon-optimized propeptide sequence only was obtained by overlap extension (SOE)-PCR. KOD Dash DNA polymerase (TOYOBO) was used to maintain the veracity of the PCR process. Restriction enzymes and T4 DNA ligase were purchased from TaKaRa and were used according to the manufacturer’s instructions. General molecular biological procedures were performed according to [34].

### Construction of expression vectors and recombinant *L. lacis* strains

The primers used in this study for the amplification of target DNA fragments are listed in Table 3. The *sp*_Usp_ gene, amplified from the chromosome of *L. lactis* MG1363 using primers P1 and P2, was cloned into *NcoI* and *SphI*-digested pNZ8149 to obtain plasmid pRF01 (Table 2). Primers P3 and P4 or P5 were used to amplify the *qk* gene encoding the propeptide and the mature QK-2 from the genome of *B. subtilis* QK02. Then, the resulting genes were digested with *KpnI* and *Xba*I and finally introduced into pRF01 to obtain plasmids pRF02 and pRF02H (Table 2). The *sp*_Usp_-*qk* was obtained from pRF02 or pRF02H using *NcoI* and *Xba*I digestion, and this fusion gene was introduced into the same digested pNZ8048 to obtain plasmids pRF03 or pRF03H (Table 2). Primers P6 and P7 were used to amplify the codon-optimized *qk*′ gene from the plasmid pUC19-qk′. The resulting fragment was introduced into pRF03H by *Kpn*I and *Xba*I digestion to obtain plasmid pRF04H (Table 2), which contained the codon-optimized *qk*′ instead of the wild-type *qk* gene. The optimized propeptide gene sequence (*pro*′) was amplified from pUC19-qk′ using primers P6 and P8 containing 15 overlapping nucleotides with the 5′ end of the mature peptide gene sequence in wild-type *qk* gene. The mature peptide gene sequence (*M*) was amplified from plasmid pRF03H using primers P9 and P10. Using primers P6 and P10, the codon-optimized propeptide only gene sequence *qkpro*′ was obtained by overlap PCR with the *pro*′ and *M* sequences as templates. Then, the *qkpro*′ was introduced into pRF03H by *Kpn*I and *Xba*I digestion to obtain plasmid pRF05H (Table 2). One or three LysM repeats can be amplified from the chromosome of *L. lactis* MG1363 using primers P11 and P12 or P13. Then, these repeats were introduced into plasmid pRF03 by *Xba*I and *Sac*I digestion to obtain plasmids pRF06H and pRF07H (Table 2), respectively. Using the chromosome of *L. lactis* MG1363, primers P11 and P14 were used to amplify three LysM repeats containing 15 overlapping nucleotides with the 5′ end of the second LysM repeat in *acmA* gene. Primers P13 and P15 were used to amplify the last two LysM repeats from the chromosome of *L. lactis* MG1363. Using primers P11 and P13, a gene fragment containing five LysM repeats was obtained by overlap PCR with the above three and two repeats as templates. The obtained five LysM repeats were also introduced into plasmid pRF03 by *Xba*I and *Sac*I digestion to obtain plasmid pRF08H (Table 2). The first LysM repeat was amplified from the chromosome of *L. lactis* MG1363 using primers P16 and P17 and was then introduced into plasmid pRF03H by *Sph*I and *Kpn*I digestion to obtain plasmid pRF09H (Table 2), which contained one LysM repeat at the 5′ end of the *qk* gene. All plasmids were sequenced at TsingKe Biological Technology (Wuhan, China) to confirm the veracity and the correct open reading frame. In addition to *L. lactis* NZ3900 containing plasmid pRF02 or pRF02H, pRF03H was introduced into NZ3900, and the transformants were selected on GM17 plates containing chloramphenicol. All plasmids containing the chloramphenicol resistance gene constructed in this study (Table 2) were introduced into *L. lactis* NZ9000 and selected as described above.

**Table 3:** Primers used in this study

Primer	Sequence^a^ (5′–3′)
P1	CTGCCATGGCAATGAAAAAAAAGATTATCTC
P2	GCGCATGCGTCGACCGCATCTTGTTTAGCA
P3	GCGGTACCGCCGGAAAAAGCAGTACAGAAAAGA
P4	TGTCTAGA *TTA*TTGTGCAGCTGCTTGTACGTTG
P5	TGTCTAGA *TTA* ***GTGGTGGTGGTGGTGGTG***TTGTGCAGCTGCTTGTACGT
P6	GCGGTACCGCTGGTAAATCATCAACTG
P7	TGTCTAGA *TTA* ***GTGGTGGTGGTGGTGGTG***TTGAGCAGCAGCTTGAACGT
P8	**AGGAACAGATTGCGC**GTATTCAAGAGCGATGTGATCTTC
P9	GCGCAATCTGTTCCTTACGGCAT
P10	TGTCTAGATTATCACTAGTGGTG
P11	TGTCTAGAGGAAATACTAATTCTGGTGG
P12	GCGAGCTC *TTA* ***GTGGTGGTGGTGGTGGTG***TGTCAGTACAAGTTTTTGA
P13	GCGAGCTC *TTA* ***GTGGTGGTGGTGGTGGTG***TTTTATTCGTAGATACTGAC
P14	**AGAAGAAGCTGAACC**TTTTATTCGTAGATACTGACCA
P15	GGTTCAGCTTCTTCTACAAATTCAG
P16	GCGCATGCGGAAATACTAATTCTGGTGG
P17	GCGGTACCTGTCAGTACAAGTTTTTGACC

^a^Underlying (single) indicates the restriction enzyme sites; Bold italics indicates the His_6_-tag sequence; Stop codons (in italics) were introduced to end the translation; Boldface indicates primer extensions necessary for overlap extension

### Induction

For the secretory expression of the free Subtilisin QK-2 or the fusion proteins, overnight cultures of the recombinant *L. lactis* NZ3900 harboring the plasmid pRF02 or pRF02H were diluted 1:25 into fresh M17 medium containing 0.5 % lactose. For other recombinant strains, overnight cultures were diluted 1:25 into fresh M17 medium containing 0.5 % glucose and 5 μg/ml of chloramphenicol. Strains were incubated at 30 °C without shaking. Nisin (Sigma) was added into the culture during the early exponential phase of growth (OD_600_ ≈ 0.5) to induce expression. To regulate the pH of the medium, 10 % culture volume of 5 N NaOH was added once at the indicated time (1, 2, or 3 h) after induction. After induction, the culture was centrifuged and both the supernatant culture and sonicated cell lysate were used for the detection and quantification of the enzyme [18].

### Production of LAB GEM particles and binding conditions

Chemical pretreatment of LAB cells, including *L. lactis* MG1363, *L. bulgaricus* ATCC 11842, *L. plantarum* ST-III, *L. casei, L. paracasei,* and *S. thermophiles,* was performed with 10 % TCA. Briefly, cells of the stationary-phase cultures were collected by centrifugation and washed twice with 0.3 volume PBS. Then, cells were resuspended in 0.2 volume of 10 % TCA solution and boiled for 30 min. Next, the cells were washed three times in PBS with vigorous vortexing. After washing, the cells were resuspended in PBS at a final concentration of 2.5 × 10^10^ GEM particles/ml, as determined using a Burker-Turk hemocytometer. GEM particles were either used immediately for binding experiments or stored at −80 °C until use.

For binding experiments, 2.5 × 10^9^ GEM particles were added to 0.5–50 ml of cell-free supernatant containing fusion proteins and incubated for 30–60 min at room temperature in an end-over-end rotator. When needed, culture medium containing the fusion proteins was concentrated prior to binding using an Amicon Ultra-15 ml (Millipore). After binding, the GEM particles were collected by centrifugation and washed three times with PBS. To test the binding efficiency, GEM particles were analyzed using immunofluorescence microscopy, western blotting or enzymatic activity analysis.

### Measure of fibrinolytic activity

Subtilisin QK-2 shows high efficiency for directly cross-linked fibrin; therefore, the fibrinolytic activity of the above samples was assayed with the standard fibrin plate method using urokinase as the standard [1]. Holes were made on the fibrin plate prepared according to the previously used method [35]. For both the cell-free culture medium containing free Subtilisin QK-2 and the GEM particles bound to the LysM-QK fusion proteins, 30 μl of the samples was dropped in the holes. Then, the plate was incubated for 18 h at 37 °C, and the fibrinolytic activity was determined by measuring the diameter of the clear zone according to the standard curve of urokinase (Sigma).

### Western blot analysis

The culture medium containing free Subtilisin QK-2 or the GEM particles bound to the fusion proteins were resuspended in SDS-PAGE loading buffer and boiled for 10 min. The proteins were separated by 12 % SDS-PAGE and transferred onto a PVDF membrane (Millipore). The membrane was blocked overnight in 5 % solution of skim milk powder in Tris-buffered saline containing 0.05 % tween-20 (TBST) and then incubated in TBST containing 1:2000 diluted mouse His-tag monoclonal antibody (Proteintech, Wuhan, China) for 2 h with gentle shaking. After washing three times with TBST buffer, the membrane was incubated with 1:6000 diluted peroxidase-conjugated goat anti-mouse IgG (H + L) (Proteintech, Wuhan, China) for 1 h. After washing, the membrane was subjected to ECL hypersensitive reagents (Pierce, USA) and then exposed and developed.

### Immunofluorescence microscopy

To further confirm that the QK and LysM fusion proteins can bind to the surface of GEM particles after the binding assay, the pellets were resuspended in 100 μl PBS containing 1 % BSA and mouse His-tag monoclonal antibody diluted 1:50. After incubation for 1 h at room temperature, the GEM particles were washed three times with 1 ml PBS. Next, they were combined with 100 μl PBS containing 1 % BSA and FITC-conjugated goat anti-mouse IgG (H + L) (Proteintech, Wuhan, China) diluted 1:100 and incubated for 1 h. GEM particles were washed three times with PBS, placed on a glass slide, air-dried and heat-fixed. Analysis was performed on a confocal microscopy (Olympus Fluoview IX70).

### Stability test

Simulated gastric juice (GJ) was prepared as previously described [36] containing 3 mg/ml pepsin (Sigma) and the pH was adjusted to 2.0 or 4.0. A 500 μl volume of 10-fold concentrated cell-free culture medium containing free Subtilisin QK-2 and 500 μl loaded MG1363 GEM particles incubated with the same concentrated cell-free culture medium containing QK-3LysM fusion protein were combined with the same volume of simulated GJ or PBS (pH 7.0; positive control), respectively, and incubated at room temperature for 30 min. Then, the mixture was used for the analysis of fibrinolytic activity as described above.

### Codon usage analysis

A graphical codon usage analyzer (http://www.gcua.schoedl.de/) [37] was used to compare the codon usage in the Subtilisin QK-2 gene *qk* with the codon usage table of *L. lactis* MG1363 (http://www.kazusa.or.jp/codon). Codon usage between 10 and 20 % for a particular codon is considered as “rather low” and below 10 % as “very low” [20].

## Additional file

10.1186/s12934-016-0478-7 Sequence alignment diagram of the wild and codon-optimized Subtilisin QK-2 gene. **Figure S2**. Optimized induction conditions. **Figure S3**. Analysis of the binding efficiency of QK-1LysM. **Figure S4**. Detection of QK-3LysM fusion protein on the surface of LAB GEM particles by immunofluorescence microscopy. **a** bright field view of loaded LAB particles; **b** merged images of the bright field view and exciting light view of loaded LAB particles. The preparations were observed through an Olympus Fluoview IX70 confocal laser scanning microscope equipped with a 100 × objective.
