# Supplementary material for: Subtilisin QK-2: secretory expression in Lactococcus lactis and surface display onto gram-positive enhancer matrix (GEM) particles
Source: Microb Cell Fact. 2016 May 12;15:80. doi: 10.1186/s12934-016-0478-7 (PMC4866291; doi:10.1186/s12934-016-0478-7)
Supplement: Supplementary file 1 — 10.1186/s12934-016-0478-7 Sequence alignment diagram of the wild and codon-optimized Subtilisin QK-2 gene. Figure S2. Optimized induction conditions. Figure S3. Analysis of the binding efficiency of QK-1LysM. Figure S4. Detection of QK-3LysM fusion protein on the surface of LAB GEM particles by immunofluorescence microscopy. a bright field view of loaded LAB particles; b merged images of the bright field view and exciting light view of loaded LAB particles. The preparations were observed through an Olympus Fluoview IX70 confocal laser scanning microscope equipped with a 100 × objective. [file 12934_2016_478_MOESM1_ESM.docx]

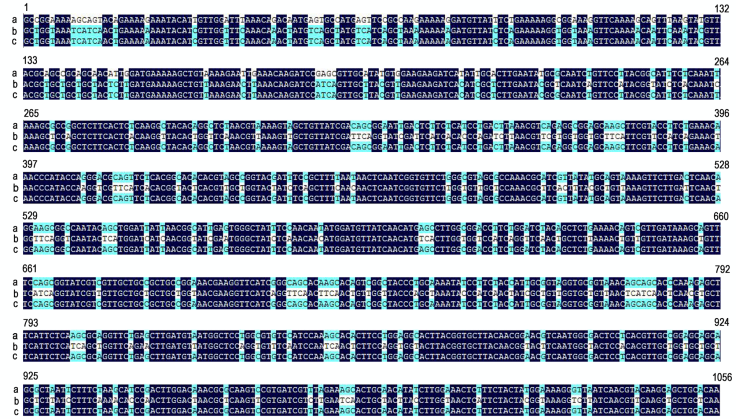


Figure S1. Sequence alignment diagram of the wild and codon-optimized Subtilisin QK-2 gene. a, wild-type gene (*qk*); b, the entire gene sequence with codon optimization (*qk’*); c, the only codon-optimized propeptide gene sequence (*qkpro’*)


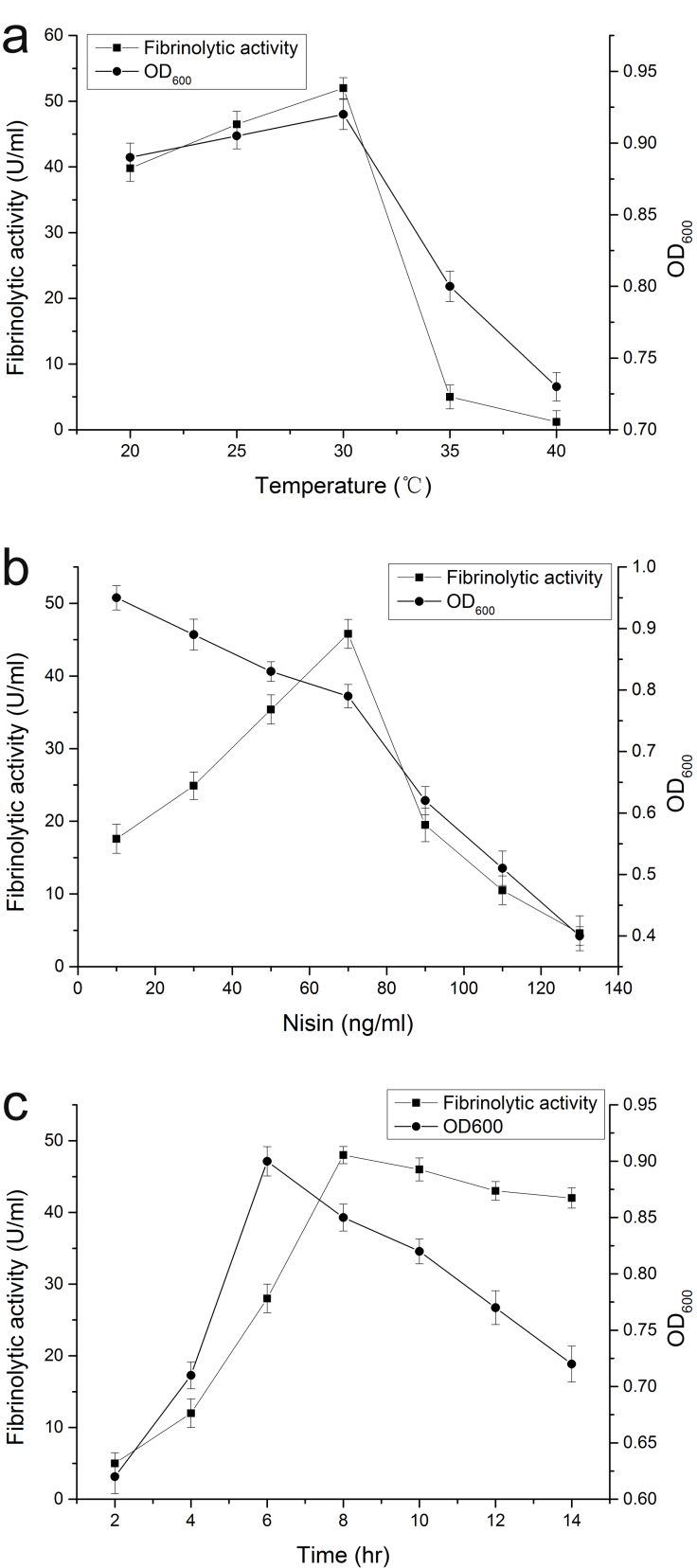


Figure S2. Optimized induction conditions. Effects of temperature (a), nisin concentration (b) and inducing time (c) to the expression of the Subtilisin QK-2 and the growth of the recombinant *L.lactis* strain. All data presented were mean ± SD of three replicate experiments

**
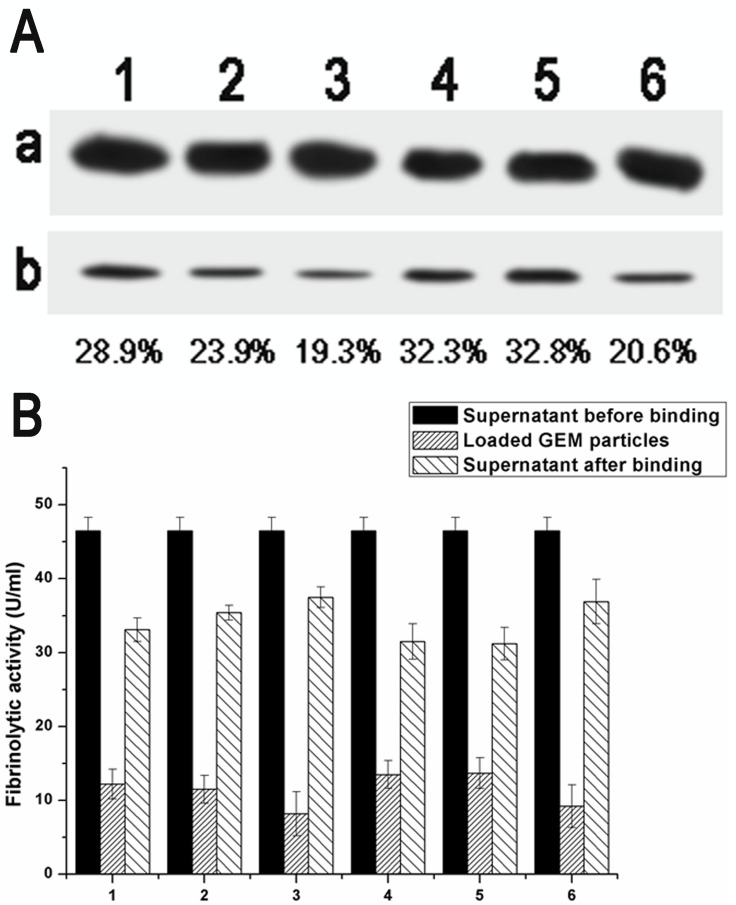
**

Figure S3. Analysis of the binding efficiency of QK-1LysM. A, western blot analysis of the culture supernatant after binding assay (a) and the QK-1LysM loaded LAB GEM particles (b); the relative binding rate is shown for each LAB GEM particle. B, fibrinolytic activity analysis of QK-1LysM before and after binding assay; all data presented were mean ± SD of three replicate experiments. 1, *L. lactis* MG1363; 2, *L. casei*; 3, *L. paracasei*; 4, *S. thermophilus*; 5, *L. bulgaricus* ATCC 11842; 6, *L. plantarum* ST-III

**
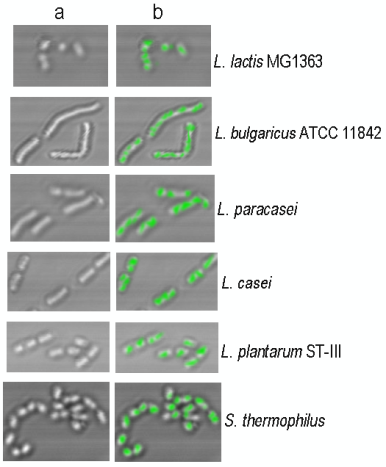
**

Figure S4. Detection of QK-3LysM fusion protein on the surface of LAB GEM particles by immunofluorescence microscopy. a, bright field view of loaded LAB particles; b, merged images of the bright field view and exciting light view of loaded LAB particles. The preparations were observed through an Olympus Fluoview IX70 confocal laser scanning microscope equipped with a 100 $\times$ objective
